# Parturition and postpartum dietary change altered ruminal pH and the predicted functions of rumen bacterial communities but did not alter the bacterial composition in Holstein cows

**DOI:** 10.3389/fvets.2022.948545

**Published:** 2022-08-26

**Authors:** Yo-Han Kim, Atsushi Kimura, Toshihisa Sugino, Shigeru Sato

**Affiliations:** ^1^Department of Large Animal Internal Medicine, College of Veterinary Medicine, Kangwon National University, Chuncheon, South Korea; ^2^Veterinary Teaching Hospital, Faculty of Agriculture, Iwate University, Morioka, Japan; ^3^Cooperative Department of Veterinary Medicine, Faculty of Agriculture, Iwate University, Morioka, Japan; ^4^The Research Center for Animal Science, Graduate School of Biosphere Science, Hiroshima University, Higashi-Hiroshima, Japan

**Keywords:** rumen bacterial community, predicted bacterial function, periparturient period, ruminal pH, Holstein cows

## Abstract

We investigated the temporal dynamics of ruminal pH and the composition and predicted functions of the rumen bacterial community in Holstein cows during the periparturient period. Eight multiparous Holstein cows (body weight; 707.4 ± 29.9 kg, parity; 3.6 ± 0.6) were used for continuous reticulo-ruminal pH measurement, among which five were also used for bacterial community analysis. Rumen fluid samples were collected at 3 weeks before and 2 and 6 weeks after parturition, and blood samples were collected 3 weeks before and 0, 2, 4, and 6 weeks after parturition. After the parturition, reduction in the 1-h mean reticulo-ruminal pH was associated with a significant (*P* < 0.05) increase in total volatile fatty acid concentration. However, with the exception of a significant change in an unclassified genus of *Prevotellaceae* (*P* < 0.05), we detected no significant changes in the compositions of major bacterial phyla or genera or diversity indices during the periparturient period. On the basis of predicted functional analysis, we identified a total of 53 MetaCyc pathways (45 upregulated), 200 enzyme commissions (184 upregulated), and 714 Kyoto Encyclopedia of Genes and Genomes orthologs (667 upregulated) at 6 weeks postpartum that were significantly (*P* < 0.05) different to those at 3 weeks prepartum. Among the 14 MetaCyc pathways (*P* < 0.05) in which pyruvate is an end product, PWY-3661 [log2-fold change (FC) = 5.49, false discovery rate (FDR) corrected *P* < 0.001] was the most highly upregulated pyruvate-producing pathway. In addition, P381-PWY [adenosylcobalamin biosynthesis II (aerobic); FC = 5.48, FDR corrected *P* < 0.001] was the second most upregulated adenosylcobalamin (Vitamin B12)-producing pathway. In contrast, PWY-2221 (FC = −4.54, FDR corrected *P* = 0.003), predominantly found in pectinolytic bacteria, was the most downregulated pathway. Our findings indicate that changes in rumen bacterial community structure are not strictly associated with transitions in parturition or diet; however, we did observe changes in reticulo-ruminal pH and the metabolic adaptation of predicted functional pathways. Consequently, predictive analysis of postpartum functional pathways may enable us to gain insights into the underlying functional adaptations of bacterial communities in Holstein cows during the periparturient period.

## Introduction

At around time that the lactation period commences, a high-grain postpartum diet is necessary to enhance rumen fermentation and meet the energy requirements of lactation (1). However, enhanced rumen fermentation is accompanied by a decline in ruminal pH, which is attributable to an increase in the production of volatile fatty acids (VFAs) or lactic acid. In turn, this contributes to increases in the occurrence and severity of ruminal acidosis and subacute ruminal acidosis (SARA) (2). SARA is defined as a condition characterized by ruminal pH below 5.6 for more than 3 h per day (3) or below 5.8 for more than 5 h per day (4). SARA has been established to be associated with a range of health problems in dairy cows, among which are feed intake depression, reduced fiber digestion, milk fat depression, an increased production of bacterial endotoxins, and inflammation (5, 6). In addition, under conditions of high-grain diet challenge, it has been observed that SARA (7, 8) or parturition (9–11) has the influences on the structure and diversity of the ruminal bacterial community.

The impact of the transition from a high-forage diet to one of high grain content on rumen microbial composition has previously been investigated in Holstein cows. For example, single SARA challenge has been found to promote reductions in the diversity and abundance of gram-negative bacteria (12–14), although adaptation to the repeated SARA challenge and a subsequent recovery associated with changes in community composition and diversity have also been detected in rumen bacterial communities (8). Similarly, changes dairy cow rumen bacterial and archaeal communities have also been identified during the transition period (11). Accordingly, these observations provide convincing evidence to indicate that the development of an acidic ruminal environment in response to the transition to a high-grain diet can have a significant influence on rumen bacterial community structure and vice versa. This transition is characterized by changes in rumen fermentation, including elevated total VFA production and altered constituent profiles, along with changes in the characteristics of rumen bacteria (8, 13).

Although these findings may be broadly applicable, they might not necessarily be equally relevant in Holstein cows during the transition period. For example, Mohammed et al. (15) have reported variability in the severity of ruminal acidosis among pre- and postpartum cows, which they attribute to variability among bacterial communities in the rumen digesta. Such findings thus highlight that cows are not equally susceptible to ruminal acidosis, and that bacterial community composition may shift independently of differences in the severity of postpartum ruminal acidosis, and does not differ substantially between the pre- and post-calving periods (15). Similarly, the findings of a further study indicate the parity (primiparous vs. multiparous) has a significant impact on the dynamics of the ruminal microbiome of cows during the transition period (10). Nevertheless, despite this variability in the rumen bacterial community, the fermentative activities of these organisms continue to be sufficiently efficient to provide the requisite energy for milk production after parturition (16), thereby highlighting the adaptability of the bacterial community (17) in overcoming these metabolic challenges.

With a view toward clarifying the mechanisms underlying this adaptability, we investigated changes in the composition and predicted function of the rumen bacterial community in Holstein cows during the periparturient period. Specifically, we examined the possible associations between these changes with co-occurring physiological changes, including those in ruminal pH, fermentation, and blood metabolites and hormones. We hypothesized that parturition and simultaneous dietary changes may induce changes in the structure and potential functions of the rumen bacterial community. Our findings may offer plausible clues that will enable us to gain a more comprehensive understanding of these relationships and provide a basis for further studies on postpartum SARA.

## Materials and methods

### Animals and group assignment

The experimental protocol devised for this study was approved by the Iwate University Laboratory Animal Care and Use Committee (A201452-2; Morioka, Japan). For the purposes of analyses, we used eight multiparous Holstein cows (body weight; 707.4 ± 29.9, parity; 3.6 ± 0.6; Supplementary Table 1), which were housed in a facility of the Hiroshima University Experimental farm (Hiroshima, Japan), in which they had free access to water throughout the study period. Prior and subsequent to parturition, these cows were fed total mixed ration containing respectively dry and lactation period diets *ad libitum* (Table 1). Dry and lactation period diets were supplied daily in two equal portions at 900 and 1,600 h. Feed compositions and amounts were based on the requirements of the Japanese Feeding Standard for Dairy Cattle.

**Table 1:** Ingredients and nutrient composition of the dry and lactation period diets on a dry matter (DM) basis.

**Items^a^**	**Dry period**	**Lactation period^b^**
**Ingredients, %**		
TMR^c^	100.0	
Lactation period concentrate^d^		41.4
Hay^e^		38.3
Corn silage		13.1
Beet pulp pellet		5.30
Feed additives^f^		1.90
**Nutrient composition, %**		
Dry matter	90.8	38.2
Total digestible nutrients	50.0	66.0
Crude protein	12.9	14.9
Neutral detergent fiber	43.4	43.0
Acid detergent fiber	26.2	25.6
Starch	13.5	15.7
Calcium	2.03	0.84
Phosphate	0.35	0.48

a All values are expressed on a dry matter basis.

b Lactation period diet was total mixed ration.

c Total mixed ration; Dry complete (Zenkoku Rakunoshiryo Co., Ltd, Tokyo, Japan) contains 50.5% total digestible nutrients (TDN), 10.5% crude protein (CP), 23.5% neutral detergent fiber (NDF), 0.5% crude fat, 14.0% crude ash, 1.8% calcium, and 0.2% phosphate on a dry matter (DM) basis.

d Plantinum mix (Zenkoku Rakunoshiryo Co., Ltd, Tokyo, Japan) contains 73.6% TDN, 17.2% CP, 18.7% NDF, 8.9% acid detergent fiber, 30.4% starch, 0.6% calcium, and 0.4% phosphate on a DM basis.

e Italian ryegrass, alfalfa, orchard, and timothy mixed hay.

f Calcium phosphate (dibasic), tricalcium phosphate, vitamin and mineral mix, sodium bicarbonate, and salt.

### Reticulo-ruminal pH, fluid, and blood analyses

Eight multiparous Holstein cows were used for continuous reticulo-ruminal pH measurement, among which five were also used for bacterial community analysis (Supplementary Table 2). Throughout the study period, reticulo-ruminal pH was measured continuously at 10-min intervals using a YCOW-S radio transmission system (DKK-TOA, Yamagata, Japan), as described by Sato et al. (18). For the purposes of calibration, a pH sensor was inserted orally and calibration was performed at standard pH values of 4 and 7 prior to and after the experiment. No changes in pH were observed during the calibration period. Rumen fluid samples were collected using an oral stomach tube in the morning before feeding 3 weeks prior to the anticipated date of parturition and 2 and 6 weeks after parturition. The initial rumen fluid sample (100–200 mL) was discarded to avoid the possible contamination of rumen fluid with saliva. Rumen fluid samples were used to analyze bacterial community and concentrations of total VFAs, individual VFAs, NH_3_-N, lactic acid, and lipopolysaccharide (LPS). Fluid samples were immediately filtered through two layers of cheesecloth and stored at −80°C until use. Blood samples were collected in the morning before feeding 3 weeks prior to the anticipated date of parturition and 0, 2, 4, and 6 weeks after parturition. Samples were collected from the jugular vein into 10-mL evacuated serum-separating tubes and heparin-containing tubes (BD Vacutainer, Franklin Lakes, NJ, USA). These were immediately centrifuged at 1,500 × *g* for 15 min at 4°C to separate the serum and then preserved at −80°C until used for analysis.

For VFA analyses, 1 mL of 25% HO_3_P in 3 *N* H_2_SO_4_ was added to 5 mL of rumen fluid. Total and individual (acetic, propionic, and butyric acids) VFAs were separated and quantified by liquid chromatography (HPLC; Shimadzu, Kyoto, Japan) using a shim-pack SCR-102H packed column (Shimadzu, Kyoto, Japan). To determine the concentration of NH_3_-N in rumen fluid, samples were analyzed based on the steam distillation method using an NH_3_-N analyzer (Kjeltec Auto Sampler System 1035 Analyzer; Tecator Inc., Höganäs, Sweden). To measure rumen LPS concentration, rumen fluid samples were centrifuged at 11,000 × *g* for 15 min at 4°C and assayed using a kinetic Limulus amebocyte lysate assay (Pyrochrome with Glucashield; Seikagaku, Tokyo, Japan) as described previously (19). For blood biochemical analyses, the concentrations of glucose (GLU), non-esterified fatty acids (NEFAs), total ketone bodies (T-KB), cholesterol (T-CHO), triglyceride (TG), total protein (TP), albumin, blood urea nitrogen, total calcium (Ca), phosphate, aspartate transaminase (AST), and γ-glutamyl transpeptidase (GGT) were measured using an Acecut automated biochemistry analyzer (Toshiba Ltd., Tokyo, Japan). Plasma growth hormone (GH), insulin (INS), glucagon (GCG), and insulin-like growth factor-1 (IGF-1) concentrations were determined based on radioimmunoassays (20).

### Isolation of DNA

Total bacterial DNA was extracted from rumen fluid samples as described by Kim et al. (21). Briefly, samples were incubated with 750 μg/mL lysozyme (Sigma-Aldrich Co., St. Louis, MO, USA) at 37°C for 90 min. This was followed by the addition of 10 μL of purified achromopeptidase (Wako Pure Chemical Industries Ltd., Osaka, Japan) at 10,000 U/mL, with the resulting mixture being incubated at 37°C for 30 min. This suspension was subsequently treated with 60 μL 1% sodium dodecyl sulfate and 1 mg/mL proteinase K (Merck Japan Ltd., Tokyo, Japan) with incubation at 55°C for 5 min, and the lysate thus obtained was extracted with three rounds of phenol/chloroform/isoamyl alcohol (Wako Pure Chemical Industries Ltd.) and chloroform (Life Technologies Japan Ltd., Tokyo, Japan). The resulting DNA was precipitated using 5 M sodium chloride and 100% ethanol, followed by centrifugation at 21,900 × *g* for 15 min at 4°C. The DNA pellet was rinsed with 70% ethanol, dried, and dissolved in Tris-hydrochloride buffer. The Purified DNA was quantified using a Biospec-nano spectrophotometer (Shimadzu Biotech, Kyoto, Japan) and stored at −80°C until used for further analyses.

### DNA sequencing

For the purposes of identifying rumen bacteria, we amplified the bacterial 16S rRNA gene using the barcoded universal primers 515F (5′-TGYCAGCMGCCGCGGTAA-3′) and 806R (5′-GGACTACNVGGGTWTCTAAT-3′) spanning the V4 hypervariable region. Polymerase chain reactions (PCR) were performed using 25-μL reaction mixture containing 12.5 μL of 2 × KAPA HiFi HotStart ReadyMix (Kapa Biosystems, Wilmington, MA, USA), 5 μL of each primer (1 μM), and 2.5 μL of template DNA (10 ng/μL). The thermal cycling conditions were as follows: an initial denaturation at 95°C for 3 min, followed by 25 cycles of 95°C for 30 s, 55°C for 30 s, and 72°C for 30 s, with a final extension of 72°C for 5 min. Amplicons were purified using AMPure XP beads (Beckman Coulter, High Wycombe, United Kingdom) according to the manufacturer's instructions. Libraries were constructed by ligating sequencing adapters and indices to the purified PCR products using the Nextera XT Sample Preparation Kit (Illumina, San Diego, CA, USA) according to the manufacturer's instructions. Paired-end sequencing (2 × 151 bp) was conducted using the Illumina MiSeq platform according to standard protocols. The sequencing data obtained have been deposited in the Sequence Read Archive of the National Center for Biotechnology Information and can be accessed *via* the SRA BioSample accession number SAMN19291604 (https://submit.ncbi.nlm.nih.gov/subs/sra/).

### Sequencing data analysis

All sequencing reads were processed using the mothur program [version 1.41.1; University of Michigan; http://www.mothur.org/wiki/; Schloss et al. (22)], following the standard operating procedure for MiSeq [https://mothur.org/wiki/MiSeq_SOP; Kozich et al. (23)] with minor modifications. To obtain a non-redundant set of sequences, unique sequences were identified and aligned against the SILVA reference database [SSURef release 128; (24)], and sequence comparisons were performed using the mothur Ribosomal Database Project training set (version 16). Sequences were clustered and classified into operational taxonomic units (OTUs) using a cutoff value of 97% similarity. All samples were standardized by random subsampling (1,269 sequences/sample) using the “sub.sample” command, resulting in the elimination of a single sample (collected at 2 weeks postpartum) due to low sequence numbers. The “summary.single” command was used to analyze the OTUs, Chao1, abundance-based coverage estimator (ACE) of species richness, and Shannon, Simpson, and Heip diversity indices.

Representative sequences for each OTU were determined using the “get.oturep” command. Sequence comparisons were performed using BLASTn (https://blast.ncbi.nlm.nih.gov/Blast.cgi) against a 16S ribosomal RNA sequence database (Bacteria and Archaea; April 2021). Representative sequences and tabulated raw count data were used to predict functional features from 16S rRNA gene data using PICRUSt2 (25) with the default pipeline. Overall functional dissimilarities among the groups were examined based on the relative abundance of MetaCyc pathways (http://MetaCyc.org), enzyme commissions (EC), and Kyoto Encyclopedia of Genes and Genomes (KEGG) ortholog counts.

### Statistical analysis

A mixed-model analysis of variance (accounting for repeated measures), using time as a fixed effect, followed by Dunnett's multiple comparison method was used to determine within-group differences. Principal component analysis (PCA) plots were constructed using the R package ggbiplot (R software version 3.3.2; R Foundation for Statistical Computing, Vienna, Austria), and non-metric multidimensional scaling (NMDS) plots were constructed in R (http://www.r-project.org; R Foundation for Statistical Computing, Vienna, Austria) using the “vegan” package. All numerical data were analyzed using Prism software (version 8.21). A *P*-value <0.05 was considered to be indicative of a significant difference and *P* < 0.10 was taken to indicate a significant tendency. Pearson correlation coefficient (r) values and significance level (*P*-value) were used to determine the relationships among rumen measurements (7-d mean ruminal pH, time with pH under 5.6, area under pH 5.6, total VFA and lactic acid concentrations, proportions of individual VFAs, and LPS concentration) and core OTUs. A heatmap was constructed using Prism software based on the Pearson correlation data. To determine whether the predicted functional pathways of the rumen bacterial community differed compared with that at 3 weeks prepartum, the raw MetaCyc, EC, and KEGG orthologs count outputs from PICRUSt2 were analyzed using DESeq2 with default parameters, after flooring fractional counts to the nearest integer (26). The inferred predicted functional pathways were considered differentially abundant (log2-fold change; FC) at a false discovery rate (FDR)-corrected significance threshold < 0.05.

## Results

### Reticulo-ruminal pH and VFAs, blood metabolites, and hormones

**Figure 1:**
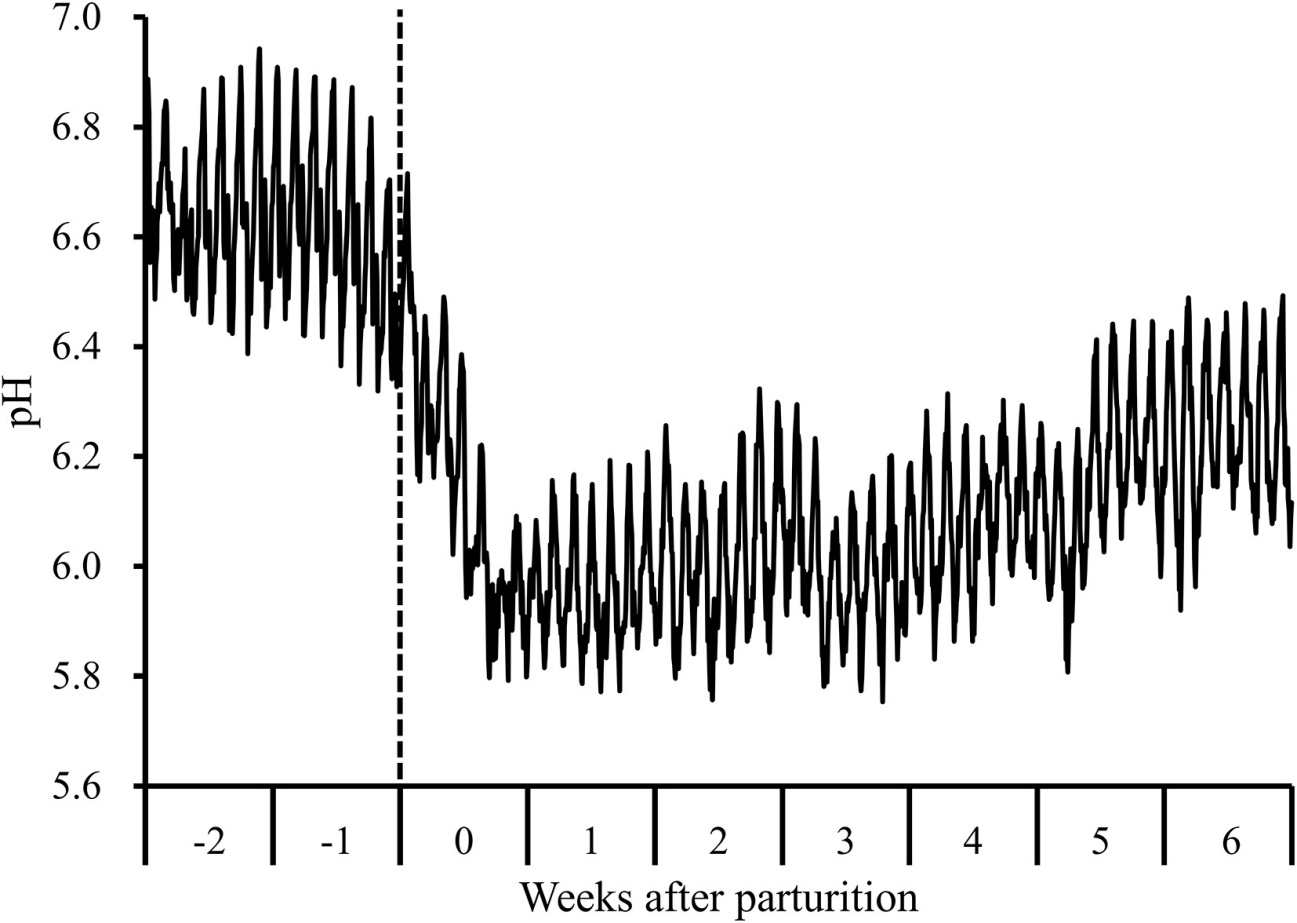
Diurnal changes in the 1-h mean reticulo-ruminal pH in Holstein cows (*n* = 8) during the periparturient period. “-2,” “-1,” “0,” “1,” “2,” “3,” “4,” “5,” and “6” denote observations at 2 and 1 weeks prepartum and 0, 1, 2, 3, 4, 5, and 6 weeks postpartum, respectively. The dotted line indicates the day of parturition.

During the periparturient period, we detected a decline in 1-h mean reticulo-ruminal pH values following parturition concomitant with the transition to a lactation period diet (Figure 1). Moreover, during this period, there were significant changes with respect to the 7-d mean reticulo-ruminal pH, and lengths of time during which pH values were <5.6 and <5.8 (*P* < 0.05). Compared with the values measured at 2 weeks prepartum, the pH values recorded at 0 to 6 weeks postpartum were significantly lower (*P* < 0.05), and the durations during which pH was below 5.6 (207 ± 28 min/d at 3 weeks postpartum) and below 5.8 (345 ± 76 and 307 ± 65 min/d at 1 and 2 weeks postpartum, respectively) were significantly higher (*P* < 0.05; Supplementary Table 3).

We also detected significant changes in total VFA concentrations (*P* < 0.05) during the periparturient period, the values of which were found to be sig6nificantly higher (*P* < 0.05) at 2 and 6 weeks postpartum compared with 3 weeks prepartum (Table 2). Similarly, we detected significant changes in the proportions of acetic and propionic acids (*P* < 0.05) during the periparturient period, which were lower and higher, respectively, compared with those determined at 3 weeks prepartum (Table 2). Consistently, we established that ruminal LPS concentration had undergone a significant change (*P* < 0.05) during the periparturient period, with higher levels being recorded at 2 and 6 weeks postpartum compared with 3 weeks prepartum.

**Table 2:** Total volatile fatty acids (VFA), proportions of individual VFAs, acetic acid to propionic acid (A/P) ratio, and NH_3_-N, lactic acid, and lipopolysaccharide (LPS) concentrations in Holstein cows (*n* = 8) during the periparturient period.

	**Weeks after**				
	**parturition**				
**Items**	**−3^b^**	**2**	**6**	**SEM**	***P*** **-value^c^**
Total VFA (mmol/dL)	5.78	8.20^a^	8.31^a^	0.86	0.009
Acetic acid (%)	68.4	62.2^a^	61.5^a^	0.62	<0.001
Propionic acid (%)	16.6	22.0^a^	22.4^a^	0.71	<0.001
Butyric acid (%)	13.2	13.4	13.7	0.55	0.644
Others (%)	1.78	2.33^a^	2.49^a^	0.10	<0.001
A/P ratio	4.11	2.87^a^	2.78^a^	0.12	<0.001
NH_3_-N	2.59	3.40	4.23	0.65	0.202
Lactic acid (mg/L)	13.4	11.5	14.6	1.51	0.365
LPS (× 10^4^ EU/mL)	3.07	7.38^a^	9.00^a^	0.76	<0.001

a Denotes significant difference (P <0.05) compared with week −3.

b -3, 2, and 6 denote 3 weeks before and 2 and 6 weeks after parturition, respectively.

c Mixed effects model ANOVA, followed by Dunnett's multiple comparison method, was used to determine within-group differences.

**Figure 2:**
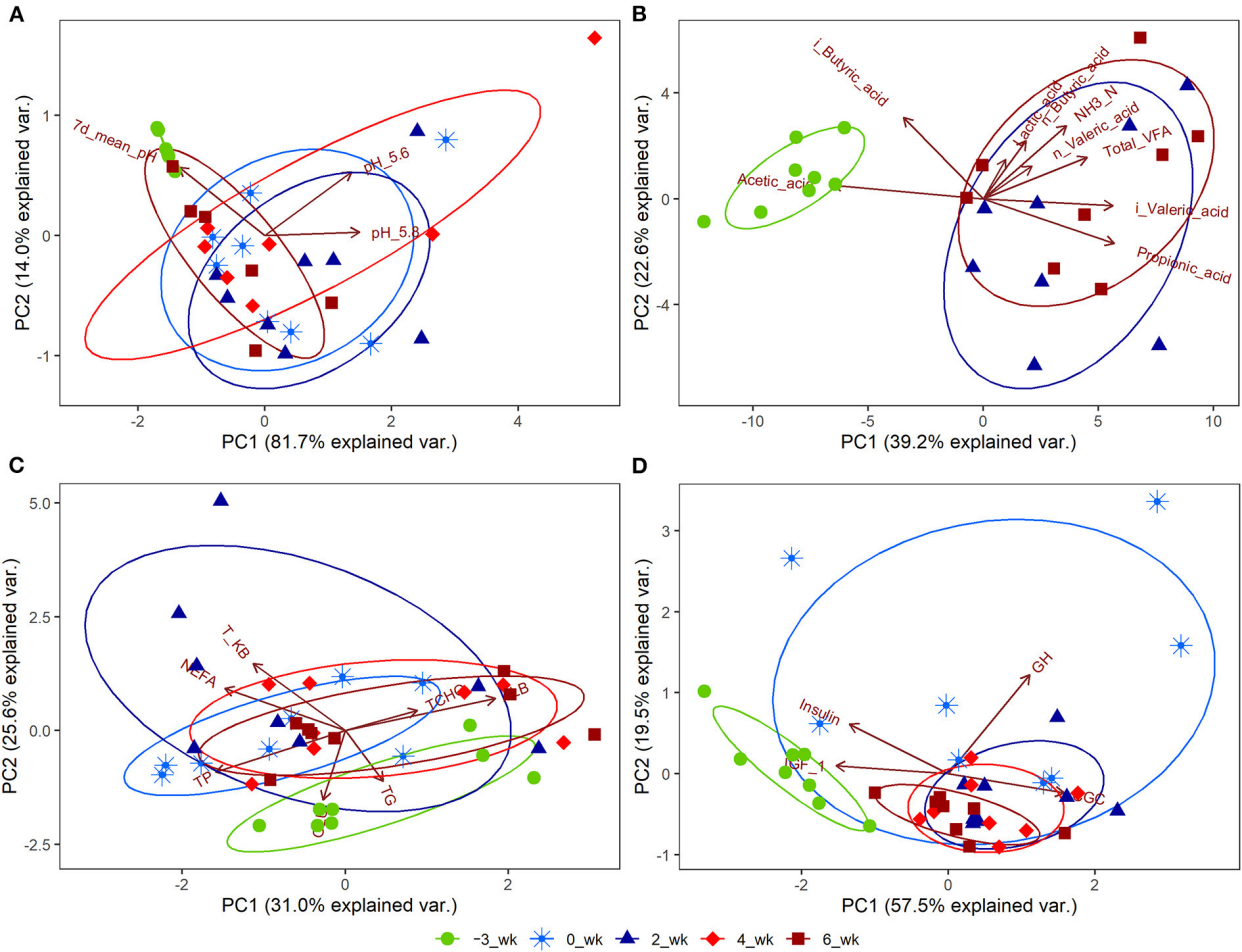
Principal component analysis (PCA) plots for Holstein cows (*n* = 8) during the periparturient period. The PCA plots were generated for **(A)** ruminal pH, **(B)** rumen fermentation measurements, **(C)** peripheral blood metabolites, and **(D)** hormones. The circle shows the correlation among the variables in each period. PC1 and PC2 represent principal components 1 and 2, respectively. 7d_mean_pH, 7-d mean reticulo-ruminal pH; pH_5.6, duration at which reticulo-ruminal pH was <5.6; pH_5.8, duration at which reticulo-ruminal pH was <5.8; GLU, glucose; NEFA, non-esterified fatty acid; T-KB, total ketone bodies; T-CHO, total cholesterol; TG, triglyceride; TP, total protein; ALB, albumin; BUN, blood urea nitrogen; Ca, calcium; iP, phosphate; AST, aspartate aminotransferase; GGT, gamma-glutamyl transferase; IGF_1, insulin-like growth factor-1; GCG, glucagon; GH, growth hormone.

Significant changes (*P* < 0.05) during the periparturient period were also detected with respect to plasma concentration of GLU, NEFA, T-KB, T-CHO, TG, TP, ALB, and Ca, and the activities of AST and GGT (Table 3). Concentrations of the hormones IGF-1, CGC, GH, and INS in the peripheral plasma were likewise significantly altered (*P* < 0.05) during the periparturient period (Table 3). Compared with 3 weeks prepartum, we detected significantly lower concentrations of IGF-1 (0, 2, 4, and 6 weeks postpartum) and INS (2 and 4 weeks postpartum; *P* < 0.05), and significantly higher concentrations of CGC (0, 2, 4, and 6 weeks postpartum) and GH (0 weeks postpartum; *P* < 0.05). PCA plots revealed 7-d mean ruminal pH (Figure 2A), the proportion of acetic acid (Figure 2B), and concentrations of GLU (Figure 2C) and IGF-1 (Figure 2D) to be the most influential factors at 3 weeks prepartum, whereas concentrations of GH were the most influential factor at 0 weeks postpartum (Figure 2D).

**Table 3:** Profiles of the peripheral blood metabolites and hormones in Holstein cows (*n* = 8) during the periparturient period.

	**Weeks after parturition**						
**Items^b^**	**−3^c^**	**0**	**2**	**4**	**6**	**SEM**	***P*** **-value^d^**
**Metabolites**							
GLU (mg/dL)	68.0	70.9	52.7^a^	58.6	62.2	3.38	0.012
NEFA (μmol/L)	0.127	0.460^a^	0.512^a^	0.228	0.170	0.052	<0.001
T-KB (μmol/L)	649.0	652.9	1,262.8^a^	889.1	929.4	106.5	0.035
T-CHO (mg/dL)	80.1	50.9^a^	98.1	132.9^a^	155.4^a^	5.73	<0.001
TG (mg/dL)	16.3	2.88^a^	5.01^a^	5.76^a^	6.08^a^	0.65	<0.001
TP (g/dL)	6.40	5.61^a^	6.28	6.31	6.70	0.74	0.011
ALB (g/dL)	5.26	4.69^a^	5.24	5.25	5.61	0.70	0.002
BUN (mg/dL)	10.5	11.2	8.08	9.51	10.2	0.82	0.056
Ca (mg/dL)	8.69	7.80^a^	9.06	8.78	9.09	0.31	0.003
iP (mg/dL)	3.55	3.54	2.46	2.81	2.91	0.68	0.067
AST (IU/L)	60.6	65.8	99.2^a^	84.4^a^	87.0^a^	7.06	<0.001
GGT (IU/L)	24.9	19.0^a^	23.9	26.2	28.5	1.55	0.002
**Hormones**							
IGF-1 (ng/mL)	83.5	36.2^a^	33.1^a^	36.6^a^	41.0^a^	4.67	<0.001
CGC (ng/mL)	0.169	0.281^a^	0.312^a^	0.314^a^	0.281^a^	0.027	0.006
GH (ng/mL)	1.96	10.8^a^	5.79	4.02	3.13	0.97	0.002
INS (ng/mL)	0.78	0.61	0.29^a^	0.41^a^	0.43	0.09	0.016

a Denotes significant difference (P <0.05) compared with week −3.

b GLU, glucose; NEFA, non-esterified fatty acid; T-KB, total ketone body; T-CHO, total cholesterol; TG, triglyceride; TP, total protein; ALB, albumin; BUN, blood urea nitrogen; Ca, calcium; iP, inorganic phosphate; AST, aspartate aminotransferase; GGT, gamma-glutamyl transferase; IGF-1, insulin-like growth factor; CGC, glucagon; GH, growth hormone; INS, insulin.

c -3, 0, 2, 4, and 6 denote 3 weeks before and 0, 2, 4, and 6 weeks after parturition, respectively.

d Mixed effects model ANOVA, followed by Dunnett's multiple comparison method, was used to determine within-group differences.

### Bacterial abundance and diversity

Although during the periparturient period we detected no significant changes (*P* > 0.1) in the relative abundances of different bacterial phyla (Table 4), at the genus level, there was a significant change in the relative abundance of an unclassified *Prevotellaceae* (*P* < 0.05), and tendencies toward significant change in the relative abundances of unclassified *Bacteroidetes* (*P* = 0.078) and *Ruminococcus* (*P* = 0.055; Table 4). Moreover, compared with 3 weeks prepartum, we observed significantly higher relative abundance of unclassified *Prevotellaceae* at 6 weeks postpartum (*P* < 0.05). At the OTU level, the total richness of all groups was determined to be 981, and a total of 37 core bacterial OTUs were identified in all samples (Supplementary Table 4). These 37 core bacterial OTUs and the positive or negative correlations among rumen measurements are shown in Supplementary Figure 1.

**Table 4:** Relative abundances of major bacterial phyla and genera (>0.05% of total sequences) and bacterial diversity indices in Holstein cows (*n* = 5) during the periparturient period.

	**Weeks after**				
	**parturition**				
**Items**	**-3^b^**	**2**	**6**	**SEM**	***P*** **-value^c^**
**Phylum**					
*Bacteroidetes*	66.1	52.8	66.3	6.84	0.127
*Firmicutes*	26.3	36.1	23.5	6.04	0.327
*Proteobacteria*	1.15	4.95	5.73	2.09	0.239
*Verrucomicrobia*	1.62	1.94	0.34	0.70	0.319
B:F ratio^d^	3.14	1.98	3.09	0.69	0.281
**Genus**					
*Prevotella*	51.5	41.0	44.1	6.03	0.317
Unclassified *Lachnospiraceae*	7.42	14.1	4.63	5.42	0.438
Unclassified *Ruminococcaceae*	8.54	8.81	7.00	2.69	0.868
Unclassified *Prevotellaceae*	2.85	3.46	15.3^a^	2.14	0.016
Unclassified *Bacteroidetes*	5.45	5.63	4.24	1.54	0.078
Unclassified *Bacteroidales*	5.98	2.25	2.13	1.33	0.352
*Succiniclasticum*	3.11	3.53	3.64	0.63	0.810
Unclassified *Gammaproteobacteria*	0.08	2.64	5.41	1.80	0.244
*Butyrivibrio*	1.16	1.77	1.31	0.35	0.459
Unclassified *Clostridiales*	1.16	1.56	1.41	0.30	0.665
*Succinivibrio*	0.95	2.22	0.25	0.90	0.261
Unclassified Subdivision	1.43	1.51	0.34	0.58	0.285
Unclassified *Firmicutes*	1.09	1.32	0.63	0.27	0.263
*Pseudobutyrivibrio*	0.61	0.82	0.81	0.16	0.587
*Ruminococcus*	0.44	0.29	1.12	0.18	0.055
*Selenomonas*	0.67	0.12	0.79	0.22	0.232
Unclassified *Clostridia*	0.54	1.03	0.03	0.44	0.276
**Diversity^e^**					
OUT	135.3	131.5	140.8	7.43	0.686
Chao1	201.0	188.1	215.8	13.2	0.373
ACE	228.4	214.3	258.3	19.6	0.309
Shannon	3.62	3.67	3.72	0.12	0.833
Simpsom	0.07	0.06	0.06	0.01	0.777
Heip	0.28	0.30	0.29	0.03	0.415

a Denotes significant difference (P <0.05) compared with week −3.

b -3, 2, and 6 denote 3 weeks before and 2 and 6 weeks after parturition, respectively.

c Mixed effects model ANOVA, followed by Dunnett's multiple comparison method, was used to determine within-group differences.

d Ratio of Bacteroidetes to Firmicutes.

e OTU, operational taxonomic unit; ACE, abundance-based coverage estimator.

### Predicted functional pathway analysis of OTUs

**Figure 3:**
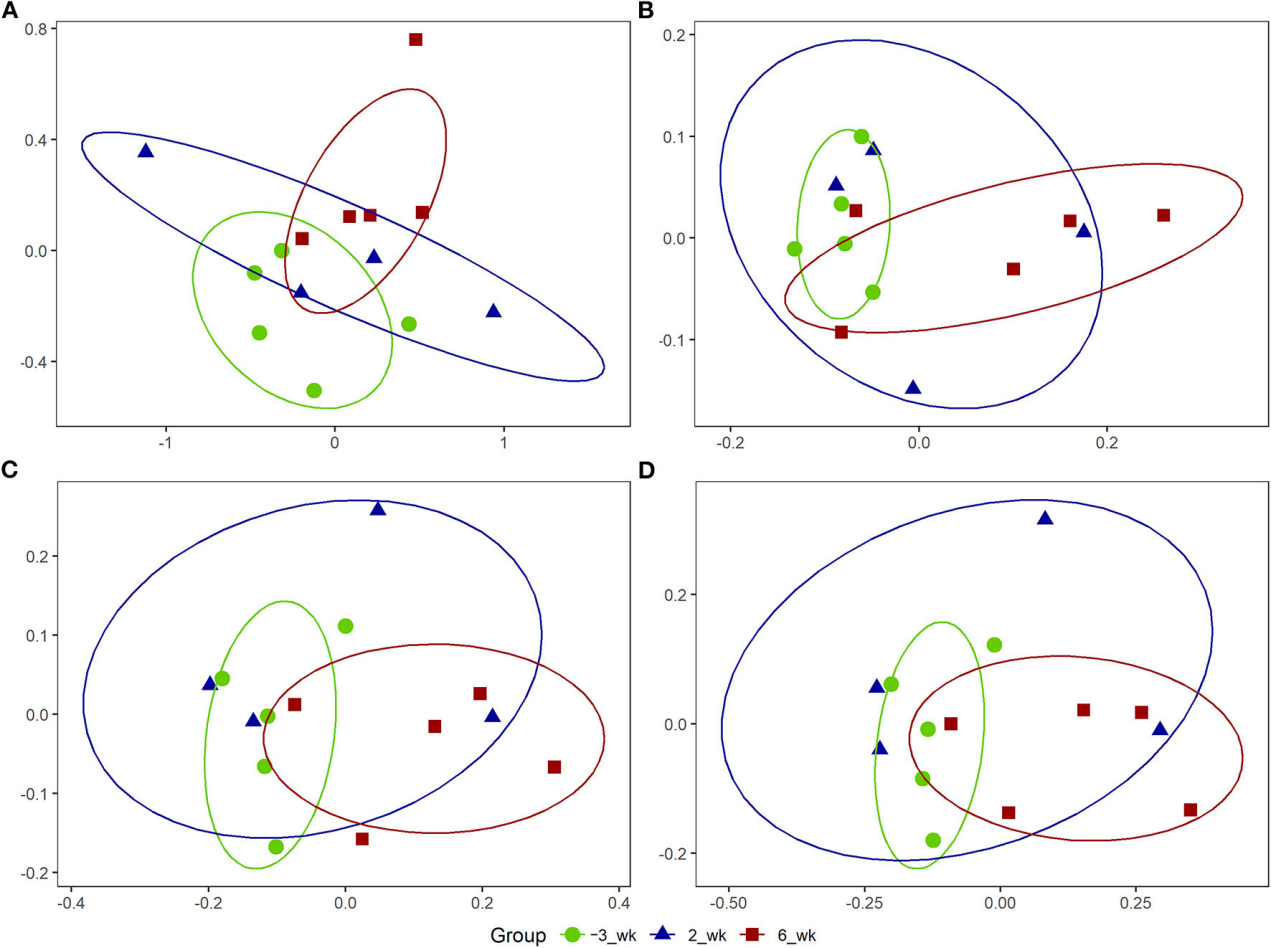
Non-metric multidimensional scaling (NMDS) plots for Holstein cows (*n* = 5) during the periparturient period. The NMDS plots were generated for **(A)** bacterial operational taxonomic units (OTUs), **(B)** MetaCyc pathways, **(C)** enzyme commissions (EC), and **(D)** Kyoto Encyclopedia of Genes and Genomes (KEGG) ortholog counts. The circle shows the correlation among the variables in each period. The stress values for OTU, MetaCyc, EC, and KEGG orthologs-based ordinations were 0.09, 0.11, 0.10, and 0.09, respectively.

Using the PICRUSt2 analysis pipeline, we identified a total of 457 MetaCyc, 2,808 ECs, and 9,955 KEGG orthologs counts. Among the analysis outputs, we established that a total of 53 MetaCyc pathways (45 upregulated; Table 5), 200 ECs (184 upregulated; Supplementary Table 5), and 714 KEGG orthologs (667 upregulated; Supplementary Table 6) at 6 weeks postpartum were significantly different (*P* < 0.05) from those at 3 weeks prepartum. However, compared with 3 weeks prepartum, we detected no significant changes among the PICRUSt2 outputs (*P* > 0.1) at 2 weeks postpartum. NMDS based on OTUs (Figure 3A) revealed that MetaCyc (Figure 3B), EC (Figure 3C), and KEGG ortholog (Figure 3D) counts at 3 weeks prepartum and 6 weeks postpartum were partitioned into two separated clusters, whereas plots constructed for 2 weeks postpartum showed a pattern of dispersed distribution. Stress values of 0.09, 0.11, 0.10, and 0.09 were obtained for out-, MetaCyc-, EC-, and KEGG ortholog-based ordinations, respectively.

**Table 5:** Predicted functional pathway analysis using the PICRUSt2 pipeline to identify MetaCyc pathways in Holstein cows (*n* = 5) during the periparturient period.

			**log2-fold**		**FDR adjusted**	
			**change** ^a^		***P*** **-value** ^b^	
**MetaCyc pathway^c^**	**Description**	**Metabolite**	**2^d^ vs. −3**	**6 vs. −3**	**2 vs. −3**	**6 vs. −3**
PWY-3661	Glycine betaine degradation I	Pyruvate	4.44	5.49	0.051	<0.001
P381-PWY	Adenosylcobalamin biosynthesis II (aerobic)	Adenosylcobalamin	4.40	5.48	0.051	<0.001
PWY-7376	Cob(II)yrinate a,c-diamide biosynthesis II (late cobalt incorporation)	Cobyrinate *a,c*-diamide	4.50	5.35	0.051	<0.001
PWY-6876	Isopropanol biosynthesis (engineered)	Propan-2-ol	4.18	5.10	0.051	<0.001
PWY-5743	3-hydroxypropanoate cycle	Acetyl-CoA	4.01	4.84	0.067	<0.001
PWY-6944	Androstenedione degradation I (aerobic)	Acetyl-CoA, succinyl-CoA, propanoyl-CoA, pyruvate	3.75	4.69	0.093	0.002
PWY-722	Nicotinate degradation I	Fumarate	4.02	4.69	0.093	0.004
PWY-5744	Glyoxylate assimilation	Propanoyl-CoA	3.65	4.58	0.119	0.003
PWY-5430	Meta cleavage pathway of aromatic compounds	Acetyl-CoA, pyruvate	3.63	4.49	0.093	0.002
PWY-6339	Syringate degradation	Pyruvate	3.45	4.12	0.117	0.004
PWY-7185	UTP and CTP dephosphorylation I	Cytidine, uridine	3.44	3.95	0.151	0.012
METHYLGALLATE-DEGRADATION-PWY	Methylgallate degradation	Pyruvate	3.27	3.87	0.151	0.012
PWY-6338	Superpathway of vanillin and vanillate degradation	Pyruvate	3.18	3.81	0.157	0.012
PWY-7097	Vanillin and vanillate degradation I	Protocatechuate	3.18	3.81	0.157	0.012
PWY-7098	Vanillin and vanillate degradation II	Protocatechuate	3.17	3.80	0.157	0.012
GALLATE-DEGRADATION-II-PWY	Gallate degradation I	Pyruvate, oxaloacetate	3.27	3.78	0.151	0.012
GALLATE-DEGRADATION-I-PWY	Gallate degradation II	Pyruvate, oxaloacetate	3.27	3.78	0.151	0.012
P184-PWY	Protocatechuate degradation I (meta-cleavage pathway)	Pyruvate	3.18	3.76	0.151	0.012
PWY-6957	Mandelate degradation to acetyl-CoA	Succinyl-CoA, acetyl-CoA	3.01	3.75	0.157	0.010
PWY-1501	Mandelate degradation I	Benzoate	3.03	3.62	0.189	0.015
PWY-5178	Toluene degradation IV (aerobic; *via* catechol)	Acetyl-CoA	3.03	3.61	0.149	0.008
PWY-6993	Nicotine degradation II (pyrrolidine pathway)	Fumarate	3.12	3.60	0.240	0.031
3-HYDROXYPHENYLACETATE-DEGRADATION-PWY	4-hydroxyphenylacetate degradation	Pyruvate, succinate	2.46	3.18	0.151	0.006
CHLOROPHYLL-SYN	3,8-divinyl-chlorophyllide a biosynthesis I (aerobic, light-dependent)	Chlorophyllide a 2	2.60	3.17	0.189	0.014
PWY-5183	Superpathway of aerobic toluene degradation	Acetyl-CoA, succinyl-CoA	2.29	3.11	0.399	0.021
PWY-7616	Methanol oxidation to carbon dioxide	CO_2_	2.45	3.09	0.197	0.012
PWY-6728	Methylaspartate cycle	Oxaloacetate	1.95	3.07	0.569	0.021
PWY-5198	Factor 420 biosynthesis II (mycobacteria)	A reduced factor 420	3.20	2.97	0.119	0.037
ARGDEG-PWY	Superpathway of L-arginine, putrescine, and 4-aminobutanoate degradation	Succinate	2.31	2.85	0.412	0.043
ORNARGDEG-PWY	Superpathway of L-arginine and L-ornithine degradation	Succinate	2.31	2.85	0.412	0.043
PWY-5531	3,8-divinyl-chlorophyllide a biosynthesis II (anaerobic)	Chlorophyllide a 2	2.26	2.84	0.399	0.038
PWY-7159	3,8-divinyl-chlorophyllide a biosynthesis III (aerobic, light independent)	Chlorophyllide a 2	2.26	2.84	0.399	0.038
P281-PWY	3-phenylpropanoate degradation	Fumarate, pyruvate	1.49	2.75	0.627	0.012
P101-PWY	Ectoine biosynthesis	Ectoine	1.88	2.72	0.407	0.015
PWY-5266	p-cymene degradation	Acetyl-CoA	1.91	2.65	0.345	0.012
PWY-5273	p-cumate degradation	Acetyl-CoA, pyruvate	1.91	2.65	0.345	0.012
PWY-5420	Catechol degradation II (meta-cleavage pathway)	Acetyl-CoA, pyruvate	1.62	2.38	0.516	0.027
PWY0-1277	3-phenylpropanoate and 3-(3-hydroxyphenyl) propanoate degradation	Acetyl-CoA	1.65	2.35	0.516	0.032
PWY-5419	Catechol degradation to 2-hydroxypentadienoate II	2-hydroxypenta-2,4-dienoate	1.52	2.27	0.605	0.042
PWY-5529	Superpathway of bacteriochlorophyll a biosynthesis	Bacteriochlorophyll a	1.00	2.27	0.869	0.012
PWY-3801	Sucrose degradation II (sucrose synthase)	β-D-fructofuranose 6-phosphate	1.50	1.95	0.439	0.038
P221-PWY	Octane oxidation	Octanoyl-CoA	1.26	1.82	0.513	0.029
PWY-1541	Superpathway of taurine degradation	Acetyl-CoA, 2-aminoacetaldehyde, sulfite, L-alanine	1.40	1.64	0.399	0.044
PWY0-1415	Superpathway of heme b biosynthesis from uroporphyrinogen-III	Protoheme	1.15	1.61	0.538	0.043
PWY-5507	Adenosylcobalamin biosynthesis I (anaerobic)	Adenosylcobalamin	0.04	0.96	0.998	0.018
PWY-5840	Superpathway of menaquinol-7 biosynthesis	Menaquinol-7	−0.03	−1.03	0.999	0.044
PWY-5838	Superpathway of menaquinol-8 biosynthesis I	Menaquinol-8	−0.05	−1.03	0.998	0.043
PWY-5897	Superpathway of menaquinol-11 biosynthesis	Menaquinol-11	−0.02	−1.05	0.999	0.044
PWY-5898	Superpathway of menaquinol-12 biosynthesis	Menaquinol-12	−0.02	−1.05	0.999	0.044
PWY-5899	Superpathway of menaquinol-13 biosynthesis	Menaquinol-13	−0.02	−1.05	0.999	0.044
P261-PWY	Coenzyme M biosynthesis I	Coenzyme M	−0.89	−1.86	0.869	0.038
PWY-7209	Superpathway of pyrimidine ribonucleosides degradation	β-alanine	−0.78	−2.22	0.869	0.038
PWY-2221	Entner-Doudoroff pathway III (semi-phosphorylative)	Pyruvate	−3.09	−4.54	0.187	0.003

a log2-Fold Change was calculated by comparisons of −3 weeks and 2 or 6 weeks after parturition.

b Adjusted P-value was calculated by false discovery rate method (27).

c Representative sequences and tabulated raw count data were analyzed using PICRUSt2 pipeline (25) to MetaCyC pathways.

d -3, 2, and 6 denote 3 weeks before and 2 and 6 weeks after parturition, respectively.

Among the MetaCyc pathways (*P* < 0.05), 14 and 11 pathways associated with the production of pyruvate and acetyl-CoA as end products, respectively, were identified as being involved in pyruvate metabolism and various metabolic process (acetyl-CoA), respectively. PWY-3661 (glycine betaine degradation I; FC = 5.49, FDR corrected *P* < 0.001) was the most upregulated pyruvate metabolism-involved pathway, and P381-PWY [adenosylcobalamin biosynthesis II (aerobic); FC = 5.48, FDR corrected *P* < 0.001] was the second most upregulated adenosylcobalamin-involved pathway, and PWY-7376 [cob(II)yrinate a,c-diamide biosynthesis II (late cobalt incorporation); FC = 5.35, FDR corrected *P* < 0.001] was the third most upregulated cobalamin-involved pathway. Among the multiple MetaCyc pathways identified for different end products, we identified three metabolic pathways for each of succinate, succinyl-CoA, propanoyl-CoA, oxaloacetate, fumarate, and chlorophyllide a 2, and three pathways for each of protocatechuate and adenosylcobalamin that were significantly upregulated (*P* < 0.05). In contrast, PWY-2221 [Entner-Doudoroff pathway III (semi-phosphorylative); FC = −4.54, FDR corrected *P* = 0.003] involved in pyruvate metabolism was identified as the most downregulated pyruvate metabolism-involved pathway. In addition, the superpathways of menaquinol-7 (FC = −1.03, FDR corrected *P* = 0.044), −8 (FC = −1.03, FDR corrected *P* = 0.043), −11 (FC = −1.05, FDR corrected *P* = 0.044), −12 (FC = −1.05, FDR corrected *P* = 0.044), and −13 (FC = −1.05, FDR corrected *P* = 0.044) biosynthesis, P261-PWY (coenzyme M biosynthesis I; FC = −1.86, FDR corrected *P* = 0.038), and PWY-7209 (superpathway of pyrimidine ribonucleosides degradation, FC = −2.22, FDR corrected *P* = 0.038) were significantly downregulated.

## Discussion

During the transition period, the diets of dairy cows are frequently modified to meet the energy requirement for milk production (1). However, abrupt transition from a low-energy dry diet to a high-energy lactating diet may render cows susceptible to rumen acidosis or SARA (16). In the present study, we detected notable reductions in 7-d and 1-h mean reticulo-ruminal pH values after parturition, concomitant with the transition to a lactation period diet, leading to a prolongation of the periods during which pH values were <5.6 and 5.8 postpartum. These observations are sufficient to diagnose SARA at 1–3 weeks postpartum, given that this condition is characterized by an extended period during which ruminal pH is below 5.6 (3) or below 5.8 (4). Furthermore, the higher total VFA and LPS concentrations, proportions of propionic and butyric acids, and a lower proportion of acetic acid during the postpartum period is consistent with the responses previously observed using high-grain diet challenge models (7, 28, 29).

A postpartum depression in reticulo-ruminal pH is considered to have a less severe impact on the blood metabolites, as no distinct differences have been detected in cows with and without SARA with respect to energy metabolism indicators (GLU, NEFA, T-KB, T-CHO, and TG) (16). In the present study, similar patterns were identified among peripheral blood indicators, such as significantly higher (NEFA, T-KB, and T-CHO) and lower (GLU and TG) values after parturition. The low levels of GLU detected after parturition tend to indicate that rumen fermentation is unable to satisfy the energy requirements of lactation, whereas elevated levels of NEFA and T-KB imply a negative energy balance due to fat mobilization for gluconeogenesis in the liver (30).

Although the occurrence of postpartum SARA is likely to attenuate the hepatic transcriptomic responsiveness to postpartum metabolic load and hormones (16), we observed no distinguishable changes from the general pattern of peripheral blood plasma hormones. Plasma GH levels, associated with the stimulation of carbohydrate and lipid metabolism and gluconeogenesis for milk production (31), were found to be elevated soon after parturition, thereby suppressing hepatic IGF-1 production *via* a negative feedback loop (32). In addition, we found that levels of INS and GCG, considered to be the primary regulators of gluconeogenesis in ruminants (33), were significantly increased and reduced after parturition, respectively. In periparturient cows, these hormones are assumed play important roles in the metabolism of a range of nutrients, and the observed changes are considered to reflect a natural adaptation designed to improve postpartum negative energy balance for milk production, leading to enhanced liver transcriptomic responsiveness to the periparturient hormonal changes (16).

With respect to ruminal bacterial community composition, unclassified *Prevotellaceae* has been identified as the only taxon that responds or adapts to dietary and reticulo-ruminal pH alterations, and in sheep, its proportional occurrence in the ruminal microbiota has been found to be higher in high-grain diet-fed animals than in those fed a low-grain diet (34). However, in the present study, we detected no significant changes in bacterial richness and diversity indices during the periparturient period. Among the core bacterial OTUs, we observed alterations in relatively few bacterial species during the periparturient period. Those that did show changes include OTU2 (*Prevotella ruminicola*), OTU37 (*Prevotella brevis*), OTU39 (*Ruminococcus albus*), OTU52 (*Prevotella melaninogenica*), and OTU57 (*Eubacterium ruminantium*), and we can thus speculate that these bacteria might play important roles in the response or adaptation to dietary and reticulo-ruminal pH changes during the periparturient period. Generally, however, it appears that the structural composition of the rumen bacterial community in Holstein cows undergoes only minor changes during the periparturient period, which is consistent with the conclusions drawn in previous studies on bacterial variability (10, 15).

In the present study, we used 16S rRNA gene sequences of representative OTUs to predict functional features using PICRUSt2. We accordingly identified a total of 53 MetaCyc pathways, 200 ECs, and 714 KEGG orthologs that were significantly upregulated at 6 weeks postpartum compared with 3 weeks prepartum, whereas in contrast, no significant changes in PICRUSt2 outputs were detected at 2 weeks postpartum, thereby tending to indicate delayed responses among members of the bacterial community. Furthermore, our NMDS analysis of OTUs revealed similar patterns among MetaCyc, EC, and KEGG orthologs, with clustering into two separate groups at 3 weeks prepartum and 6 weeks postpartum and a dispersed distribution at 2 weeks postpartum.

MetaCyc is a universal database describing scientifically verified metabolic pathways and enzymes from all domains of life (35), and recent studies have frequently used this database to identify functional changes in ruminal bacteria metabolism (36, 37). We therefore assessed the predicted functional pathways of the rumen bacterial community by focusing on MetaCyc pathways, given that changes in PICRUSt2 outputs showed similar patterns during the periparturient period. We accordingly established that 26.4 and 20.8% of the identified MetaCyc pathways produce pyruvate and acetyl-CoA as an end product, respectively. Pyruvate is a central intermediate metabolite that can enter numerous pathways supplying energy to bovine ruminal cells (2), and rumen microbial species generate energy *via* the oxidation of pyruvate to VFAs (38, 39). Within the rumen, hexose (glucose, fructose, and galactose) glycolysis and the pentose (xylose) phosphate pathway are the metabolic pathways used to convert plant carbohydrates to pyruvate (38). Acetyl-CoA, the product of 11 of the identified MetaCyc pathways, is an intermediate metabolite in a range of ATP-generating metabolic pathways (39).

Glycine betaine, a component of the most highly expressed functional pathway (PWY-3661) identified in the present study, is a naturally occurring compound produced as a metabolic by-product of choline oxidation (40). Supplementation of the diets of primiparous lactating dairy goats with glycine betaine has been shown to induce the production of higher levels of short-chain fatty acids in milk and also higher milk production and levels of milk fat during late lactation (41). The second most highly expressed functional pathway (P381-PWY) produces adenosylcobalamin (a biologically active form of Vitamin B_12_) as an end product, which is synthesized exclusively by mainly anaerobic microorganisms (42, 43). Gut-synthesized adenosylcobalamin is absorbed through the gastrointestinal tract, and transported *via* the blood to body tissues and fluids including the liver, muscles, and milk (44). Intramuscular injections of vitamin B_12_ have been shown to increase the yields energy-corrected milk, and levels of milk solids, fat, and lactose in early lactation primiparous dairy cows (45). The third most highly expressed functional pathway identified in the present study (PWY-7376) produces cobyrinate *a,c*-diamide. This pathway, referred to as the “late cobalt insertion” pathway (46), is associated also associated with vitamin B_12_. Cobalt is a key ionic component the vitamin B_12_, in the structure of which, it is bound to the center of tetrapyrrole-derived macrocycle (46), and consequently, a source of cobalt is essential for the formation of vitamin B_12_ by rumen microorganisms (47). The PWY-7376 pathway would thus appear to represent an important step involved in the incorporation of cobalt ions in the structure of vitamin B_12_, and is accordingly connected with the sub-pathway P381-PWY in the rumen bacterial community. Collectively, the findings of our predictive analyses would thus tend to indicate that the high expression of these three functional pathways, along with that of the multiple pathways identified for certain other products, might represent a delayed adaptive response of the bacterial community to an abrupt transition in the diet of Holstein dairy cows after parturition.

In addition to the upregulated responses of the aforementioned pathways, we also identified a number of bacterial functional pathways characterized by downregulated responses to dietary transition and the associated low reticulo-ruminal pH. Among these, the most notable is the Entner–Doudoroff pathway, which in gram-negative bacteria, certain gram-positive bacteria, and archaea plays a prominent role in the catabolism of glucose to pyruvate (48). In this regard, previous studies have demonstrated high activity of 2-keto-3-deoxy-6-phosphogluconate aldolase (the final enzyme of the Entner–Douforoff pathway) in rumen pectinolytic bacteria, including *Butyrivibrio fibrisolvens* 787 and *Prevotella ruminicola* AR29, and is present among bacteria grown on a pectin substrate, although not in those grown on a glucose substrate (49). Although we failed in the present study to detect any significant changes among bacteria in the genera *Prevotella* and *Butyrivibrio*, we did, nevertheless, establish that the transition from a high-forage to low-forage (high-grain) diet might induce the suppression of pectinolytic activity (Entner–Douforoff pathway) among the predicted responses of the rumen bacterial community. With respect to the PWY-7209 pathway, degradation of purines and pyrimidines, releasing small amount of NH_3_, is a part of the urea recycling system in lactating dairy cows (50). Although the P261-PWY was postulated based on methane-producing bacteria, downregulation of this pathway is consistent with reduced methanogenesis associated with the consumption of a high-grain diet compared with the high-forage diet feeding in beef steers (51). Furthermore, long-chain menaquinone species (5 to 13), referred to as Vitamin K_2_ are synthesized by bacteria (52), and we found that the bacterial function of menaquinol-7, 8, 11, 12, and 13 biosynthesis was reduced in response to transition to a high-grain diet after parturition.

## Conclusion

On the basis of our observations, it would appear that parturition and postpartum dietary changes do not induce any marked alterations in rumen bacterial community of Holstein cows during the periparturient period. However, dietary transition may promote changes in the functional pathways of certain bacteria to satisfy energy requirements and/or facilitate adaptation to a change in diet and the associated lower reticulo-rumen pH in these cows.

## Data availability statement

The datasets presented in this study can be found in online repositories. The names of the repository/repositories and accession number(s) can be found in the article/Supplementary material.

## Ethics statement

The animal study was reviewed and approved by Iwate University Laboratory Animal Care and Use Committee.

## Author contributions

TS and SS: conceptualization. AK, TS, and SS: investigation. Y-HK: data curation and writing—original draft preparation. Y-HK, AK, and SS: writing—review and editing. All authors have read and agreed to the published version of the manuscript.

## Funding

This research was financially supported by a grant from the Ito Foundation, Japan.

## Conflict of interest

The authors declare that the research was conducted in the absence of any commercial or financial relationships that could be construed as a potential conflict of interest.

## Publisher's note

All claims expressed in this article are solely those of the authors and do not necessarily represent those of their affiliated organizations, or those of the publisher, the editors and the reviewers. Any product that may be evaluated in this article, or claim that may be made by its manufacturer, is not guaranteed or endorsed by the publisher.

## Supplementary material

Supplementary Figure 1 Correlation analyses between relative operational taxonomic unit (OTU) abundance and rumen measurements. Cells are colored based on Pearson correlation analyses, where blue and red represent negative and positive correlations, respectively. ^*^ denotes a significant correlation (*P* < 0.05) between rumen measurements and OTUs. 7-d mean pH, 7-d mean reticulo-ruminal pH; pH <5.6, period during which reticulo-ruminal pH was <5.6; pH <5.8, period during which reticulo-ruminal pH was <5.8; EU, endotoxin units.

Supplementary Table 1 Changes in body weight, body condition score, dry matter intake, milk yield, and milk fat composition in Holstein cows (*n* = 8) during the periparturient period.

Supplementary Table 2 The profile of sample collection in experimental cows (*n* = 8) during the periparturient period.

Supplementary Table 3 The 7-d mean reticulo-ruminal pH and duration of times during which pH was <5.6 and 5.8 in Holstein cows (*n* = 8) during the periparturient period.

Supplementary Table 4 Relative abundances and taxonomic classification of operational taxonomic units (OTUs; shared by all samples) in Holstein cows (*n* = 5) during the periparturient period.

Supplementary Table 5 Predicted functional pathway analysis using the PICRUSt2 pipeline to identify Enzyme Commissions (EC) in Holstein cows (*n* = 5) during the periparturient period.

Supplementary Table 6 Predicted functional pathway analysis using PICRUSt2 pipeline to identify Kyoto Encyclopedia of Genes and Genomes (KEGG) orthologs in Holstein cows (*n* = 5) during the periparturient period.
