# Supplementary material for: Parturition and postpartum dietary change altered ruminal pH and the predicted functions of rumen bacterial communities but did not alter the bacterial composition in Holstein cows
Source: Front Vet Sci. 2022 Aug 26;9:948545. doi: 10.3389/fvets.2022.948545 (PMC9458962; doi:10.3389/fvets.2022.948545)
Supplement: Supplementary Figure 1 — Correlation analyses between relative operational taxonomic unit (OTU) abundance and rumen measurements. Cells are colored based on Pearson correlation analyses, where blue and red represent negative and positive correlations, respectively. * denotes a significant correlation (P < 0.05) between rumen measurements and OTUs. 7-d mean pH, 7-d mean reticulo-ruminal pH; pH <5.6, period during which reticulo-ruminal pH was <5.6; pH <5.8, period during which reticulo-ruminal pH was <5.8; EU, endotoxin units. [file Data_Sheet_1.DOCX]

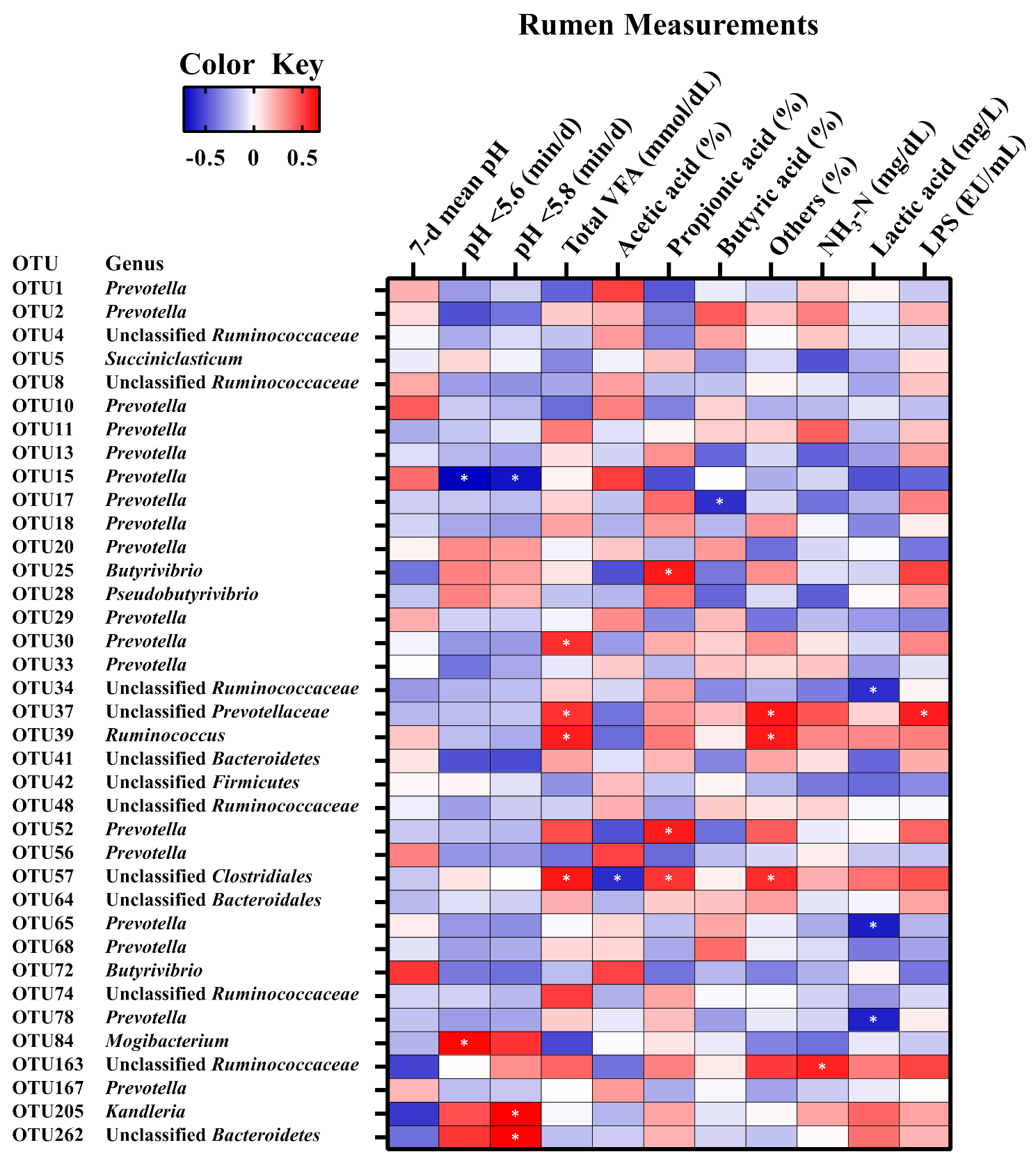


**Supplementary Figure S1**. Correlation analyses between relative operational taxonomic unit (OTU) abundance and rumen measurements. Cells are colored based on Pearson correlation analyses, where blue and red represent negative and positive correlations, respectively. * denotes a significant correlation (*P* < 0.05) between rumen measurements and OTUs. 7-d mean pH, 7-d mean reticulo-ruminal pH; pH <5.6, period during which reticulo-ruminal pH was <5.6; pH <5.8, period during which reticulo-ruminal pH was <5.8; EU, endotoxin units.
